# Is the concept of mammalian epigenetic clocks universal and applicable to invertebrates?

**DOI:** 10.3389/fgene.2025.1633921

**Published:** 2025-08-08

**Authors:** Ryszard Maleszka

**Affiliations:** Research School of Biology, Australian National University, Canberra, ACT, Australia

**Keywords:** epigenomics, PWWP domain, social insect, honey bee, epigenetic diversity, ageing

## Abstract

Certain aspects of animal ageing can be quantified using molecular clocks or machine learning algorithms that are trained on specific omics data, with epigenetic clocks based on DNA methylation (DNAm) garnering the most attention. While the accuracy of epigenetic clocks has been established in mammals and several vertebrates, their applicability to invertebrates, which comprise 97% of all animal species, remains largely theoretical. In this context, we consider whether the relationship between chronological clocks, biological clocks, and DNA methylation is ancestral and evolutionarily conserved, potentially making it relevant beyond the vertebrate lineage. Evolutionary comparisons may help us determine whether epigenetic clocks are inherent mechanisms implemented during ageing or simply reflect the progressive erosion of epigenomic marks. These comparisons could also reveal the likely generality of the results from one type of epigenetic clock to another. We emphasise the substantial biological differences between invertebrates and mammals, all of which must be considered when evaluating the universality of epigenetic clocks. We conclude that mammalian-style DNAm epigenetic clocks are unlikely to be applicable to most invertebrates. We propose that quantitative approaches to ageing in non-vertebrate organisms should be specifically tailored to leverage the molecular mechanisms and distinct biology of different lineages.

## Introduction

*“Choosing the right organism for one’s research is as important as finding the right problems to work on (Sydney Brenner, Nobel Lecture, 2002).*”

Over the past decade, research has documented age-related changes in the mammalian DNA methylome and identified specific cytosine methylation sites that, when combined, can be used to measure chronological and biological age. This concept is known as the DNA methylation (DNAm) clock or, more broadly, the epigenetic clock (Teschendorff and Horvath, 2025; Field et al., 2018). It is considered a valuable biomarker for distinguishing between healthy and unhealthy ageing and for assessing disease risks, such as late-onset cancers. Although some methylated CpG dinucleotides have been deemed potentially causal, it is not clear at present if the DNA methylation differences used for predicting age contribute to the ageing process or are just bystanders reflecting spatio-temporal erosion of flexible epigenomic marks (Ying et al., 2024; Yang et al., 2023; Bertucci-Richter and Parrott, 2023).

As with many intriguing discoveries in one group of organisms, numerous follow-up studies have been conducted to explore whether epigenetic clocks based on DNA methylation can measure chronological or biological age in non-mammalian species. While the premise of epigenetic clocks seems to hold in several vertebrates tested so far (Zoller et al., 2024), evidence supporting this process in invertebrates is contentious and remains hypothetical (Liu et al., 2025).

Since more than 97% of all animal species are invertebrates (May 1988), we ask whether the relationship between chronological clocks, biological clocks, and DNA methylation is ancestral and evolutionarily conserved and, thus, might be applicable outside the vertebrate lineage.

We highlight the important biological differences between mammals and invertebrates that must be considered in this context. These differences include their distinct DNA methylation machinery, with multiple examples of invertebrates missing this level of epigenomic modification (Kucharski et al., 2023), their varying and elaborate life cycles, and the lineage-specific epigenetic nature of relatively short lifespans (Maleszka and Kucharski, 2022; Yu et al., 2019; Heinze and Giehr, 2021). The long-lived reproductive females in eusocial insects are presented as an example of epigenetically controlled environmental influence on the genomic capacity to generate contrasting organismal outcomes, including extended longevity (Heinze and Giehr, 2021; Miklos and Maleszka, 2011; Blacher et al., 2017).

We conclude that the mechanisms driving mammalian epigenetic clocks, which are based on DNA methylation, cannot be directly applied to most invertebrates. Short-lived organisms represent a fundamentally different evolutionary strategy, and the idiosyncrasies influencing their ageing may not be comparable to those in longer-lived vertebrate models and to humans. We propose that innovative approaches, not confined to DNA methylation alone, are essential for developing algorithms that measure age in invertebrates based on molecular changes.

## What makes the transferability of mammalian DNAm epigenetic clocks to invertebrates problematic?

Historically, invertebrate research has been at the forefront of biological science, paving the way for advancements in mammalian studies, including those related to human health. Model organisms like *Drosophila melanogaster* and *C. elegans* are regarded as gold standards in many spheres of discovery (Bertile et al., 2023; Miklos and Maleszka, 2000). However, in the context of methylomics, specifically DNAm epigenetic clocks, the process is reversed, with data from mammals being utilised to inform research on invertebrates. This approach carries inherent risks, as transferring data and concepts between mammals and evolutionarily older lineages can be problematic (Maleszka and Kucharski, 2022; Maleszka et al., 1998; Hardison, 2016; Setola and Roth, 2003).

Vertebrates and invertebrates represent two extremely diverse classes of animals. When considering DNAm epigenetic clocks, several aspects of their respective biology must be considered.

First, while the accuracy for predicting chronological age is ±1–3 years (Zhang et al., 2019), impressive for mammals, this error range encompasses the entire lifespan of most invertebrates, such as nematodes and insects. Although some species, including sponges, jellyfish, and annelids, exhibit remarkably long lifespans, sometimes referred to as “immortal”, their unusual longevity is not necessarily linked to the presence of DNA methylation machinery (see discussion below).

Second, the age-related changes in DNA methylation are subtle, typically involving only 2%–5% of methylated CpG dinucleotides over many decades (Teschendorff and Horvath, 2025; Seale et al., 2024). Thus, in addition to the timeframe that does not apply to most invertebrates, discovering rare predictive CpGs in their sparsely methylated genomes poses an entirely different challenge. Another feature of mammalian epigenetic clocks that is not compatible with most invertebrates’ lifespans is the 24-h periodicity with fluctuations up to 5 years within a single day (Koncevičius et al., 2024).

Third, various advanced tools, such as Illumina bead array technology and sophisticated algorithms trained on specific omics, can achieve the precision of DNA methylation changes observed in mammals (Bell et al., 2019). These technologies have not yet been developed for use in other lineages.

Finally, the solution to the problem of the universality of epigenetic clocks is not limited to methyl-cytosines; it must also include a better understanding of demethylation and methyl-binding systems, both of which are under-researched in invertebrates (Cramer et al., 2017; Wojciechowski et al., 2014).

## Longevity, ageing and DNA methylation in invertebrates

DNA methylation is an evolutionarily ancient feature found in basal metazoans (Dabe et al., 2015; Ying et al., 2022), including sponges, which are regarded as the most ancient extant metazoan lineage that diverged from other metazoans over 600 million years ago (Yi et al., 2015). However, there is no clear correlation between lifespans, senescence, and the presence of DNA methylation biochemistry across the tree of life (Table 1) (Maleszka and Kucharski, 2022; Lewis et al., 2020). Among the most ancient metazoans, the distribution of the DNA methylation toolkit is not uniform (Sarkies, 2022). In the four basal phyla, Porifera (sponges), Cnidaria (sea anemones, corals, and jellyfish), Ctenophora (comb jellies), and Placozoa, some species possess the core enzymes of the DNA methylation toolkit, namely, both types of DNA methyltransferases (DNMTs), DNMT1 and DNMT3, and ten-eleven translocation (TET) methylcytosine dioxygenases, while others do not. Notably, this characteristic is not linked to longevity or senescence. Long-lived sponges and all comb-jellies possess this enzymology, whereas all four extant species of Placozoa do not have this level of epigenomic modification (Wedd et al., 2022). In the phylum Cnidaria, the “immortal” jellyfish *Turritopsis dohrnii* lacks both DNMTs and TET enzymes, whereas another Cnidarian, *Hydra vulgaris*, which exhibits no apparent senescence, possesses DNMT1 and DNMT3, as well as TET.

**TABLE 1:** Longevity and DNA methylation systems in selected organisms. These examples illustrate the lack of correlation between maximum lifespans and the presence of highly diverse DNA methylation machinery across various taxa.

Lifespans	Phyla	Phylum		*Species*	DNAm toolkit
Unlimited or extreme longevity	Invertebrates	Cnidaria		*Turritopsis dohrnii* (Immortal jellyfish)	No
				*Hydra vulgaris* (common hydra)	Yes
		Placozoa		All four extant species	No
		Porifera		Sponges	Yes^a^
		Molluscs		*Arctica islandica* (ocean clam)	Yes^b^
		Annelids		*Lamellibrachia satsuma* (tube worm)	Yes
Short lifespans		Arthropods^c^	Insects	*Diptera* (flies, mosquitoes)	No
				*Tribolium castaneum* (flour beetle)	Partial (DNMT1 only)
				*Apis mellifera* (honey bee)	Yes
				*Danaus Plexippus* (monarch butterfly)	Partial (DNMT1 only)
				*Dendroctonus ponderosae* (mountain pine beetle)	No
			Nematoda	*Caenorhabditis elegans* (eutelic nematode)	No
				*Trichuris suis* (parasitic whipworm)	Partial (DNMT3 only)
				*Plectus sambesii* (free-living nematode)	Partial (DNMT1 only)
≥200 years	Chordata	Vertebrates		*Somniosus microcephalus* (Greenland shark)	Yes
				*Balaena mysticetus* (bowhead whale)	Yes
Several months to several years		Tunicates		*Ciona intestinalis*	Yes
				*Boryllus schlosseri* (star tunicate)	No

^a^ e.g. *Xestospongia muta* (giant barrel sponge).

^b^ Although the genome of this species has not been sequenced, all available Mollusc genomes encode single-copy genes encoding DNMT1/3 and TET.

^c^ There are examples of arthropods with extended lifespans, including reproductive females (queens) in eusocial insects, and species with long periods of pre-adult larval stages. Also, the American lobster can live for up to 150 years. In all these exceptional cases, the DNA methylation system is present.

Insects have the most diverse complements of DNMTs with no apparent relationship to their lifespans, developmental strategies, or life cycles (Kucharski et al., 2023; Bewick et al., 2017). All dipterans (e.g., flies, mosquitoes) have lost their DNA methylation toolkits, and DNMT-less species are found across other large orders, such as Coleoptera (beetles), Strepsiptera (twisted-wing parasites), and Neuroptera (net-winged insects), among others. While there is nothing different or unusual about the lifespans of insects without DNA methylation, DNAm epigenetic clocks cannot be used to measure their ageing. In insects that methylate their genomes, most have only DNMT1, although the copy number varies from 1 to 3. In these species, DNMT1 likely possesses both *de novo* and maintenance capacities, although its catalytic activity has not been experimentally investigated. In approximately 25% of insects, both DNMT1 and DNMT3 have been identified, with variable copy numbers (Kucharski et al., 2023). While these proteins show sequence similarity to their mammalian counterparts, their enzymatic activities have been tested *in vitro* only in the honey bee *Apis mellifera* (Wang et al., 2006). Interestingly, in the order Hymenoptera (bees, wasps, and ants), multiple copies of DNMT1 and DNMT3 have been identified, along with an unusual duplication of the functionally essential PWWP domain in DNMT3, which binds to H3K36me2 and H3K27me chromatin modifications, in contrast to the mammalian PWWP domain that only binds to H3K36me (Kucharski et al., 2023; Maleszka, 2024).

Insect DNA methylation toolkits exemplify how evolution can create diverse epigenomic layers that confer lineage-specific advantages. This raises several important questions: What benefits do additional DNMTs or duplicated PWWP domains in DNMT3 offer to many species? How does a partial toolkit with only one DNMT1 and no DNMT3 function in most insects? It is essential to consider the unique ways in which insects and other invertebrates employ either full or partial DNA methylation toolkits, especially when developing non-mammalian epigenetic clocks.

Understanding the diverse roles of DNA methylation across all taxonomic groups is essential for advancing invertebrate methylomics. Genome defence and regulatory function are considered the ancient roles of cytosine methylation, with the regulatory aspect lost in species with low rates of cellular turnover, which may include eutelic organisms with a fixed cell number (Maleszka and Kucharski, 2022; Regev et al., 1998). In Hydra and several other Cnidarians, DNA methylation predominantly targets transposons, especially the evolutionarily youngest ones (Ying et al., 2022). There is a notable preference for methylation to occur in longer and more highly active genes. Interestingly, as transposons age, their levels of methylation tend to decline. This decline helps mitigate the potentially harmful mutagenic effects associated with CpG methylation. The relationship between the extent of DNA methylation and the content of transposons, observed in Cnidaria and other invertebrates, may provide valuable insights into the context of ageing.

The mosaic distribution of DNMT1 and DNMT3 in invertebrates (Kucharski et al., 2023; Bewick et al., 2017; Engelhardt et al., 2022) suggests that their roles vary significantly across different species, and these functions are only beginning to be understood in selected cases. For instance, in a clonal ant species, the knockout of DNMT1 leads to a decrease in DNA methylation and results in sterility (Ivasyk et al., 2023). In honey bees, silencing DNMT3 through RNA interference (RNAi) results in a higher proportion of females exhibiting the queen phenotype (Kucharski et al., 2008). Recent research has identified that a transposon-derived microRNA, known as miR-3721, post-transcriptionally regulates DNMT3 (Jiang et al., 2025). Treating larvae with agomir-3721 produces phenotypic effects similar to those observed in the RNAi experiment. Additionally, the role of DNA methylation in regulating gene expression and alternative splicing has been demonstrated in several studies (Li-Byarlay et al., 2013; Foret et al., 2012; Flores et al., 2012; Xu et al., 2021; Elango et al., 2009). However, knowledge about demethylation processes in invertebrates remains limited, with only one study showing such a role for a single TET enzyme in honey bees (Wojciechowski et al., 2014). In the insect with no DNA methylation, *D. melanogaster*, a TET homolog seems to mediate N6-methyladenine demethylation and 5 mC demethylation in DNA and mRNA, respectively (Zhang et al., 2015).

Invertebrates generally have only a single MBD protein, MBD2/3, that does not always contain appropriate residues for selectively binding methylated DNA. However, the sponge *Ephydatia muelleri* has genes for each of the NuRD core components, including an EmMBD2/3 that selectively binds methylated DNA. NMR analyses reveal a remarkably conserved binding mode. These data support a model in which the MBD2/3 methylation-dependent functional role emerged with the earliest multicellular organisms and has been maintained to varying degrees across animal evolution (Cramer et al., 2017).

## Can long-lived insects be used to develop models for invertebrate epigenetic clocks?

Maximum lifespans can vary greatly among different species, even those that are closely related (Cohen, 2018; Jones et al., 2014). Several factors influence the lifespans of animals, including body size, metabolism, and environmental conditions, which are considered the most significant. Generally, smaller animals with higher metabolic rates tend to have shorter lifespans. However, the environment is crucial in affecting lifespans, even among closely related species.

While the vast majority of insects live for a few weeks to several months, cicadas, which are known for their periodic emergence, can live underground for 17 years as nymphs, and splendour beetles can reach 25–30 years living as larvae in a host tree. In such cases, the pre-adult stages exist in a comfortable niche that is fully protected from environmental insults, highlighting the importance of the environment in longevity.

Although transferring data from model organisms to humans is not straightforward, especially when considering the influence of social environments on healthy ageing (Mason et al., 2017), eusocial insects, such as ants, termites, and honey bees, may offer valuable insights into the epigenetics of longevity (Promislow et al., 2022; Omholt and Amdam, 2004; Keller and Jemielity, 2006). These insects live in highly organised societies that affect their lifespan, with social cues enabling older individuals to exhibit more youthful behaviour (Amdam, 2011). While queens of eusocial insects do not display the same behavioural flexibility as non-reproductive workers, they can reach an age of 20–30 years with continuing high fecundity (Kramer et al., 2016). This is a compelling example of how different epigenetic interpretations of a single genome can result in contrasting phenotypic outcomes, including variations in ageing (Miklos and Maleszka, 2011). The social structures within these insect communities support an evolutionary theory of ageing, as purely mechanistic explanations for senescence do not account for the relationship between social structure and ageing outcomes (Mason et al., 2017).

**FIGURE 1:**
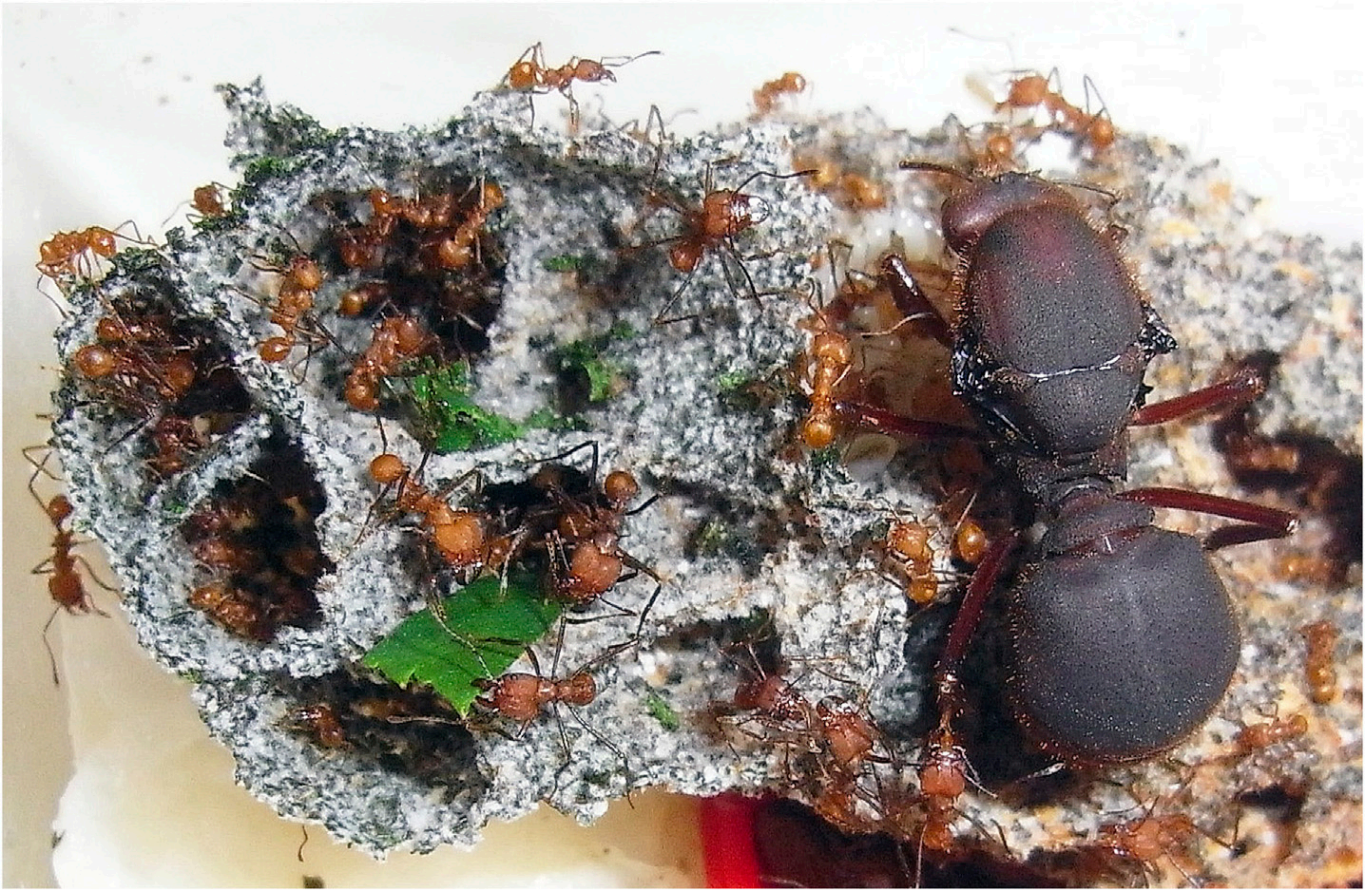
A young leaf-cutter queen (*Atta sexdens*) and her founding fungus garden with workers. The queen is approximately 8 months old. In the field, adult nests reach 8 years, but this species can live up to 18 years in the lab, underscoring the impact of environmental stressors like climatic seasonal changes or food availability/quality on longevity. The workers’ lifespan is 10–20 times shorter. Photo courtesy of Daniela Römer and Flavio Roces.

When considering female castes of eusocial insects as experimental models for designing approaches to study DNAm epigenetic clocks, it is essential to account for their elaborate life cycles. In holometabolous insects, which represent about 80% of all insect species, the preadult stages are often as long, or even longer, than the adult stages. The larval feeding stage is particularly susceptible to external influences. Most larval tissues, except for certain parts of the nervous system and small clusters of progenitor cells known as imaginal discs (ImDs), are destroyed during metamorphosis (Maleszka and Kucharski, 2022; Beira and Paro, 2016). During the pupation stage, these undifferentiated but committed ImDs give rise to the adult structures. These progenitor cells represent the only continuity at the cellular level in these insects and may provide valuable insights into the epigenomic changes that occur during post-embryonic development. Significant life span extension observed in eusocial queens can be achieved by different feeding, which in honey bees involves a potent diet called royal jelly (Miklos and Maleszka, 2011). Additionally, these highly fecund females spend their lives in a protected environment, and it is known that their longevity is negatively affected when they are outside of their optimal conditions. For example, leaf-cutter queens can live up to 8 years in the wild, but in a laboratory setting, they can reach up to 18 years (Figure 1). This scenario underscores the adverse effects of environmental stressors, such as seasonal climatic changes and fluctuations in food availability or quality, on longevity. The developmental differentiation between functionally sterile female workers and reproductive queens is epigenetically controlled, particularly in honey bees, where it is dependent on DNA methylation (Kucharski et al., 2008), and may involve other epigenomic regulatory layers, in particular histone modifications and microRNAs (Jiang et al., 2025; Wojciechowski et al., 2018; Dickman et al., 2013; Ashby et al., 2016). However, the complexities of the interactions between environmental factors and the epigenome remain largely unexplored. Understanding these epigenetic processes could be highly valuable in the context of epigenetic clocks.

## The research conducted thus far does not support epigenetic DNAm clocks in invertebrates

So far, only one report, deposited in bioRxiv, claims the discovery of an epigenetic clock in an insect, namely, the parasitic wasp *Nasonia vitripennis* (Brink et al., 2023). However, in this study’s published, peer-reviewed version, the authors have toned down their claims, including the title, opting for an exploration of “… the ageing methylome in the model insect, *N. vitripennis*” (Brink et al., 2024). Indeed, their approach using 19 pre-selected CpGs was questioned as likely to lead to overfitting to the experimental samples (Liu et al., 2025). In another Hymenopteran, the honey bee (*Apis mellifera*), no significant loss of age-related DNA methylation has been found in several genes using ultra-deep amplicon sequencing (Kucharski and Maleszka, 2020). These results are particularly significant because, unlike most insects, Hymenoptera possess a complete DNA methylation toolkit that is more comparable to the mammalian toolkit (Kucharski et al., 2023).

Regarding other arthropods, age-dependent DNA methylation changes have been reported in two species: the aquatic crustacean *Daphnia magna* (Hearn et al., 2021) and the European lobster *Homarus gammarus* (Fairfield et al., 2021). The *D. magna* epigenetic clock was built using 12 CpGs and two age groups. However, in a recent study, attempts to reproduce age-related changes in *Daphnia* across multiple life stages and to construct an epigenetic clock using machine learning models were unsuccessful (Liu et al., 2025). The authors conclude that, due to the overall low DNA methylation levels and lack of robust age-associated methylation changes, age-associated methylomics in *D. magna* should focus on environmental factors to reveal methylation dynamics (Liu et al., 2025).

The study of European lobsters is particularly important because it can enhance the accurate assessment of their population dynamics, which is essential for sustainable fisheries management. Notably, these lobsters have a relatively long lifespan, 31 years for males and 54 years for females, allowing epigenetic clocks to address the challenges posed by their indeterminate growth and the shedding of their exoskeleton throughout their lives. The initial research, which examined ribosomal DNA methylation in lobsters aged between 0 and 51 months, established a linear correlation between age and rDNA methylation. This correlation was successfully applied to individuals whose ages were unknown (Fairfield et al., 2021).

## Can other epigenetic modifications and cellular mechanisms be utilised to construct ageing clocks with comparable accuracy to DNA methylation clocks?

Given the apparent difficulties of using DNA methylation as an ageing indicator in invertebrates, it may be prudent to consider other cellular mechanisms as candidates for such a role. An interesting suggestion was made in a recent study on a 117-year-old female whose longevity was associated with a significant shortening of telomeres (Santos-Pujol et al., 2025). The authors conclude that, in light of her good health, chromosomal attrition acted more like a chromosomal clock than a predictor of age-related diseases. This is a testable hypothesis that may benefit invertebrate studies.

With the expanding assortment of epigenomic modifications and their potential roles in various cellular and organismal contexts, it is becoming possible to consider other modifiers. One key area of focus is the modifications occurring at the histone level. Unlike DNA methylation, which is not universally present, histone and chromatin modifications are found in all eukaryotic organisms. Notably, the age-related remodelling of heterochromatin and the loss of chromatin associated with hypomethylation suggest a relationship between these two regulatory layers. Importantly, ageing-dependent remodelling of heterochromatin and chromatin loss, associated with hypomethylation, suggests a connection between these two regulatory layers (Ciccarone et al., 2018). A recent analysis of multiple tissues has shown that the dynamics of seven histone marks during human ageing yielded results comparable to those of DNA methylation age predictors (de Lima Camillo et al., 2025). The findings revealed a trend characterised by a loss of modifications linked to heterochromatin and an increase in marks associated with euchromatin, indicating an overall decline in epigenetic regulation with age. This area of research is expected to grow significantly within mammalian biomedical studies and may also provide valuable insights for similar inquiries in other lineages.

Other epigenomic mechanisms, such as RNA modifications, are starting to gain attention in invertebrate research (Zhang et al., 2015; Jiao and Palli, 2024). Their ubiquitous presence and roles in cellular mechanisms (Wang et al., 2022) suggest that their involvement in ageing processes could be leveraged to design RNA-based epigenetic clocks akin to DNA methylation (DNAm) epigenetic clocks. In this context, the somewhat unexpected role of the enzyme TET in demethylating N6-methyladenine in DNA and 5-methylcytosine in RNA in the fly *D. melanogaster*, which lacks a DNA methylation toolkit (Zhang et al., 2015), suggests that a more extensive repertoire of DNA and RNA modifications might be considered for quantifying ageing, even in species lacking DNA methylation.

However, various methods to quantify biological ageing may not necessarily measure the same thing and ultimately yield different results, suggesting that comparative analyses may pose a significant challenge (Belsky et al., 2018).

## Ageing as an epigenetic, environmentally influenced process

In vertebrates, studies on convergent evolution have established that the genetic architecture of longevity-related genes is an important factor influencing the differing lifespans of closely related species with similar genomes. For instance, genetic diversity plays a role in the contrasting lifespans of two species of rougheye fish: the rockeye (*Sebastes aleutianus*), which lives for about 200 years, and its close relative, the blue rockfish (*Sebastes mystinus*), which typically lives for only 26 years (Kolora et al., 2021). These two species inhabit distinct environmental niches that present different survival challenges. The long-lived *S. aleutianus* resides in very deep, cold waters (−0.3°C–5.0°C) near the seabed, often within caves, while *S. mystinus* lives near the surface. The relative contributions of genetics and environment to prolonged longevity have been established for many organisms. For example, the protective environment of a eusocial insect colony contributes to the long lives of epigenetically generated reproductive queens. In humans, both genetics and environmental factors (the exposome) influence health, with the exposome shaping unique patterns of disease and mortality, independent of genetic factors (Argentieri et al., 2025). A computer model supports the evolutionary role of the environment in tuning lifespans. It shows that a limited lifespan can be detrimental to an individual in the short term but beneficial to their distant descendants. The model also predicts that the most beneficial lifespan varies with the environmental conditions (Werfel et al., 2015).

This interplay between contributing factors indicates that the epigenetic basis of longevity may have different implications across species (Mariani et al., 2010). Additionally, ageing exemplifies biological degeneracy, where different mechanisms can yield similar functional outcomes (Edelman and Gally, 2001; Maleszka et al., 2014). For example, in the ageing human brain, various cellular malfunctions can lead to similar symptoms known as dementia, and in species lacking a DNA methylation toolkit, alternative epigenomic layers are responsible for controlling genome-environment interactions (Mason et al., 2017; Maleszka et al., 2014). Conserved pathways, such as the insulin/IGF-1 signalling pathway and the mTOR node, regulate lifespan and can be studied in model organisms like yeast, nematodes, flies, and mice. However, it is crucial to approach studies at the epigenetic level and within the context of molecular clocks for ageing cautiously, interpreting results through a broader comparative framework. Furthermore, incorporating research on non-traditional and unusual species, while integrating both mechanistic and demographic studies, is essential (Cohen, 2018; Tian et al., 2017).

## Conclusion

There are no straightforward answers when it comes to the quantitative approaches to the biology of ageing, and research strategies should reflect this complexity. This difficulty is especially apparent when analysing the epigenetic machinery, which varies considerably between mammals and invertebrates. Significant challenges for invertebrate epigenetic clocks include computational methodology, particularly in areas such as interpretation, cell-type heterogeneity, and the adoption of emerging single-cell techniques. Technology is no longer a limiting factor, and with new developments emerging frequently, all epigenetic research will inevitably adapt to these changes. These new methodologies aim to establish guidelines for the rigorous development of interpretable epigenetic clocks at both cell-type and single-cell resolutions. Exploring additional ideas, predictions, and even speculations about epigenetic clocks in invertebrates would be valuable within the framework of modern theories of ageing and the concept of “genes for ageing.”

## Data Availability

The original contributions presented in the study are included in the article/supplementary material, further inquiries can be directed to the corresponding author.
